# Comprehensive Analysis of the Incidence and Survival Patterns of Lung Cancer by Histologies, Including Rare Subtypes, in the Era of Molecular Medicine and Targeted Therapy: A Nation-Wide Cancer Registry-Based Study From Taiwan

**DOI:** 10.1097/MD.0000000000000969

**Published:** 2015-06-19

**Authors:** Jeffrey.S. Chang, Li-Tzong Chen, Yan-Shen Shan, Sheng-Fung Lin, Sheng-Yen Hsiao, Chia-Rung Tsai, Shu-Jung Yu, Hui-Jen Tsai

**Affiliations:** From the National Institute of Cancer Research, National Health Research Institutes (JSC, L-TC, C-RT, H-JT); Department of Internal Medicine, National Cheng Kung University Hospital, Tainan (L-TC, H-JT); Department of Internal Medicine, Kaohsiung Medical University Hospital, Kaohsiung (L-TC, S-FL, H-JT); College of Medicine, Kaohsiung Medical University (S-FL); Institute of Molecular Medicine, National Cheng Kung University (L-TC); Department of Surgery, National Cheng Kung University Hospital (Y-SS); Institute of Clinical Medicine, National Cheng Kung University, Tainan (Y-SS); Department of Internal Medicine, E-Da Hospital, College of Medicine, I-Shou University (S-YH); Cancer Center, Kaohsiung Medical University Hospital (S-JY); Graduate Institute of Medicine, College of Medicine, Kaohsiung Medical University (H-JT), Kaohsiung, Taiwan.

## Abstract

Lung cancer is the third most common cancer in the world and has the highest cancer mortality rate. A worldwide increasing trend of lung adenocarcinoma has been noted. In addition, the identification of epidermal growth factor receptor (EGFR) mutations and the introduction of EGFR inhibitors to successfully treat EGFR mutated non–small cell lung cancers are breakthroughs for lung cancer treatment. The current study evaluated the incidence and survival of lung cancer using data collected by the Taiwan Cancer Registry between 1996 and 2008. The results showed that the most common histologic subtype of lung cancer was adenocarcinoma, followed by squamous cell carcinoma, small cell carcinoma, large cell carcinoma, neuroendocrine tumors, lymphoma, and sarcoma. Overall, the incidence of lung cancer in Taiwan increased significantly from 1996 to 2008. An increased incidence was observed for adenocarcinoma, particularly for women, with an annual percentage change of 5.9, whereas the incidence of squamous cell carcinoma decreased. Among the subtypes of lung cancer, the most rapid increase occurred in neuroendocrine tumors with an annual percentage change of 15.5. From 1996–1999 to 2005–2008, the 1-year survival of adenocarcinoma increased by 10% for men, whereas the 1-, 3-, and 5-year survivals of adenocarcinoma for women increased by 18%, 11%, and 5%, respectively. Overall, the incidence of lung cancer has been increasing in Taiwan, although the trends were variable by subtype. The introduction of targeted therapies was associated with a significantly improved survival for lung adenocarcinoma in Taiwan; however, more studies are needed to explain the rising incidence of lung adenocarcinoma. In addition, it is important to investigate the molecular pathogenesis of the various subtypes of lung cancer to develop novel therapeutic agents.

## Abstract 2

Supplemental Digital Content is available in the text

## INTRODUCTION

Approximately 1,824,701 new cases (age-standardized incidence rate = 23.1 per 100,000) of lung cancer are diagnosed worldwide each year, making lung cancer the third most common cancer in the world.^1^ Lung cancer has the highest mortality rate (age-standardized mortality rate = 19.7) among all cancers, with approximately 1,589,925 persons dying of lung cancer worldwide each year.^1^ According to the 2011 Taiwan Cancer Registry Annual Report, lung cancer was the third most common cancer for both men (N = 6938, age-standardized incidence rate = 38.0 per 100,000) and women (N = 4121, age-standardized incidence rate = 22.0 per 100,000) and ranked first and second in cancer mortality among women (13.9 per 100,000) and men (30.2 per 100,000), respectively.^2^

The 3 major histologic subtypes of lung cancer are adenocarcinoma, squamous cell carcinoma, and small cell carcinoma. Using data from Europe, North America, and Oceania extracted from Cancer Incidence in Five Continents, Lortet-Tieulent et al^3^ reported a declining trend among men for most subtypes of lung cancer except adenocarcinoma. Adenocarcinoma is the most common lung cancer subtype among women, and its incidence among women has been increasing.^3^ The declining prevalence of smoking may explain the decrease in the incidence of lung squamous cell carcinoma in men. However, the increase in the incidence of lung adenocarcinoma without a concurrent rise in the incidence of lung squamous cell carcinoma in women suggested different etiologies for the development of different subtypes of lung cancer. Although the incidence rates of the major subtypes of lung cancer have been well studied, there have been few reports on the incidence or mortality rates of rare primary tumors of the lung, including large cell carcinoma, sarcoma, lymphoma and neuroendocrine tumors (NETs). In our previous study, we noticed an increasing trend in the incidence of NETs across all body sites, particularly for the rectum and lung/bronchus.^4^ In addition, the male-to-female ratio of NETs is smaller than that of squamous cell carcinoma but larger than that of adenocarcinoma, which also supports the existence of different etiologies for the various subtypes of lung cancers. Therefore, investigating the epidemiologic patterns of the rare lung cancer subtypes along with those of the major subtypes may provide new insights regarding the pathogenesis of lung cancer.

Although the prognosis of lung cancer is generally poor, it is not the same for all subtypes. Before the FDA's approval of epidermal growth factor receptor (EGFR) inhibitors to treat non–small cell lung cancer (NSCLC) in 2003, the overall survival for advanced lung cancer patients treated with chemotherapy was similar for Asian and Western countries.^5–8^ However, the prognosis of lung cancer became different after the introduction of EGFR inhibitors. Lung cancers with EGFR mutations are more likely to be adenocarcinoma and occur more often in women, East Asians, and nonsmokers.^9–11^ Lung cancers with EGFR mutations have been associated with a more favorable clinical response to EGFR inhibitors.^12^ In phase II or III trials, EGFR inhibitors were more effective for pretreated NSCLC patients from East Asian populations than for patients from US or European populations.^13–16^ Furthermore, first-line EGFR inhibitors achieved good response and prolonged the survival of advanced lung cancer patients with EGFR mutations.^12,17,18^ Therefore, genetic profiling may help determine the treatment strategy and predict the prognosis for NSCLC patients.

According to the International Agency for Research on Cancer, the incidence trends of lung cancer differs by histologic subtypes, particularly for adenocarcinoma and squamous cell carcinoma in Western countries.^3^ The survival of lung cancer patients has been significantly improved by EGFR inhibitors, particularly for Asian patients with lung cancer harboring specific EGFR mutations; however, most data published to date have been hospital-based or clinical trial-based and mostly on NSCLC. Analyses of the incidence, distribution, and survival of patients with small cell lung cancer, large cell lung cancer, lung sarcoma, lung lymphoma, and lung NETs have been limited. In addition, there is no nationwide population-based study of lung cancer from Asia. In this study, we used data collected by the Taiwan Cancer Registry (TCR) to analyze the incidence, distribution, and survival of lung cancer in Taiwan. The aim of this study was to comprehensively evaluate the trends in the incidence and survival of the different subtypes of lung cancer and the effect on lung cancer survival after the introduction of targeted therapies for lung cancer in Taiwan.

## MATERIALS AND METHODS

This study was approved by the research ethics committee of the National Health Research Institutes, Taiwan. Individual consent is not required because the data are deidentified and reported in group form.

### Description of the Taiwan Cancer Registry

Data used for the current analysis were ascertained from the TCR and the Death Registry Database housed in The Collaboration Center of Health Information Application, Ministry of Health and Welfare, Taiwan. The TCR, which covers approximately 97% of cancer cases occurring in Taiwan, was established in 1979 to track the incidence and mortality rates of cancer in Taiwan.^9^ The percentage of death certificate-only cases (DCO%) (the best DCO% = 0) and the percentage of morphologically verified cases (MV%) (the best MV% = 100) are often used to assess the data quality of a cancer registry.^19^ The DCO% of the TCR decreased from 14.2% in 1996 to <2% in 2005 and thereafter.^9^ The MV% of the TCR ranged from 87.5% in 2002 to 90.8% in 2009.^9^ These indices indicate that the data quality of the TCR is similar to that of the other well-established cancer registries across the world.^20,21^

### Identification of the Incident Lung Cancer Cases

The incident cases of lung cancer diagnosed in Taiwan between January 1, 1996, and December 31, 2008, were identified from the TCR using the topography codes of the International Classification of Diseases for Oncology, Field Trial Edition (ICD-O-FT) (for those diagnosed between January 1, 1996, and December 31, 2001) or the International Classification of Diseases for Oncology, Third Edition (ICD-O-3) (for those diagnosed after January 1, 2002). The histologic subtypes of lung cancer were assigned according to the morphology (M) codes and included adenocarcinoma, squamous cell carcinoma, small cell carcinoma, large cell carcinoma, NET, sarcoma, lymphoma, and others. For NET, we adopted the M codes used by Hauso et al ^22^ and included: 8240 (carcinoid tumor), 8241 (enterochromaffin cell carcinoid), 8242 (enterochromaffin-like cell tumors), 8243 (goblet cell carcinoid), 8244 (composite carcinoid), 8245 (adenocarcinoid), 8246 (neuroendocrine carcinoma), 8249 (atypical carcinoid), 8013 (large cell neuroendocrine carcinoma), and 8574 (adenocarcinoma with neuroendocrine differentiation).

### Statistical Analysis for the Age-Standardized Incidence Rates and Incidence Trends of Lung Cancer

The crude annual incidence rates of lung cancer in Taiwan from 1996 to 2008 were analyzed by histologic subtypes, sexes, and age groups, using the annual populations reported by the Directorate-General of Budget, Accounting, and Statistics of Taiwan (http://www.dgbas.gov.tw). Age-standardized incidence rates were generated using the 2000 WHO standard population. The annual percentage change (APC) was estimated to assess the incidence trends of lung cancer overall and by histologic subtypes using linear regression: log(rate_y_) = b_0_ + b_1_y, with log(rate_y_) = natural log of incidence rate in year y. APC = (e^b1^–1)x100. A significant change in incidence trend was indicated by a *P* < 0.05.

### Survival Analysis of Lung Cancer

Data from the TCR were linked to the Death Registry Database to determine the vital status and date of death for each lung cancer case. The 1-, 3- and 5-year survival of lung cancer overall, by sex, histologic subtype and time period were calculated using the life-table method. A Cox proportional hazards regression model was used to estimate the hazard ratio (HR) and 95% confidence interval (CI) of lung cancer death associated with histologic subtype, age, sex, stage and the time period of diagnosis. In addition to examining the survival of lung cancer diagnosed during 1996–2008, survival analyses were also performed for 3 separate periods: 1996–1999, 2000–2004, and 2005–2008. The 3 time periods were defined based on the following factors: there was a revision of the NET classification by the World Healthcare Organization in 2000, and in Taiwan, Gefitinib, an EGFR inhibitor, was approved as a third-line treatment for lung adenocarcinoma in 2003 and the reimbursement for such use was approved by the Bureau of National Health Insurance (BNHI) in November of 2004. Comparing the survival of lung adenocarcinoma cases diagnosed in 2005–2008 to those diagnosed before 2005 allowed us to evaluate the overall benefit of the EGFR inhibitor for treating lung adenocarcinoma.

## RESULTS

### Age-Standardized Incidence Rates

**FIGURE 1:**
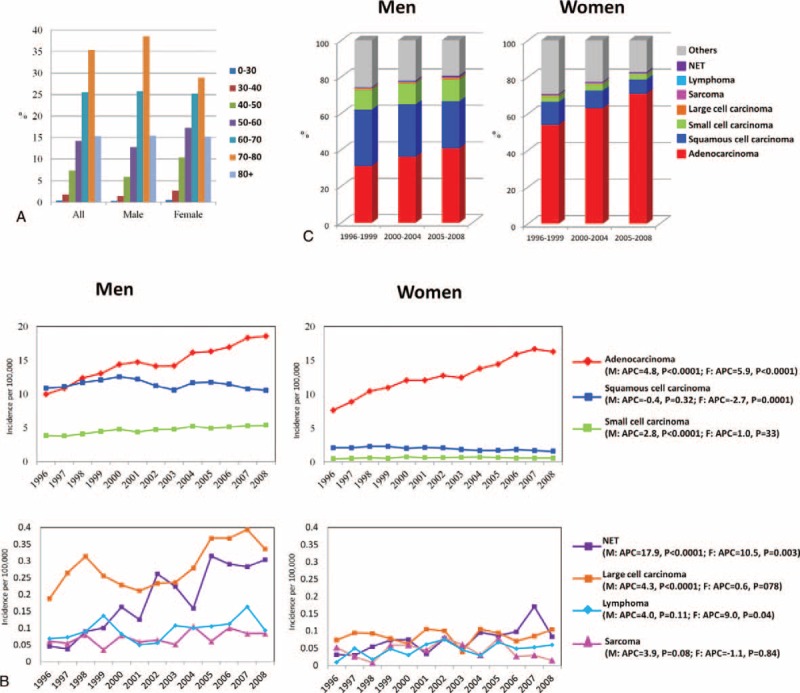
(A) Age distribution of lung cancer patients overall and by sex; (B) Incidence trend of lung cancer in Taiwan from 1996–2008 by subtype and sex; (C) Distribution of lung cancer by subtype, sex, and time period.

A total of 97,031 newly diagnosed lung cancer cases were recorded in the TCR from January 1, 1996, to December 31, 2008, with 65,493 (67.5%) men and 31,538 (32.5%) women. The mean age was 67.8 for all subjects, 68.5 for men, and 65.7 for women. The incidence rate of lung cancer increased with age, with those aged between 70 and 80 years old accounting for the largest proportion (35.4%); 76.3% of the lung cancers were diagnosed at age ≥60 years (Figure 1A). The age-standardized incidence rate of lung cancer in Taiwan increased from 26.0 per 100,000 in 1996 to 32.1 per 100,000 in 2008 (APC = 1.7, *P* < 0.0001) (Supplementary Table 1, http://links.lww.com/MD/A300). Men consistently had a higher incidence rate of lung cancer than women but the male-to-female ratio of the incidence rate decreased from 2.16 in 1996 to 1.91 in 2008. For men, the incidence rate of lung cancer was 34.8 per 100,000 in 1996, increasing to 42.4 per 100,000 in 2008 (APC = 1.6, *P* = 0.0002). For women, the incidence rate of lung cancer increased from 16.09 per 100,000 in 1996 to 22.20 per 100,000 in 2008 (APC = 2.6, *P* < 0.0001), which was more rapid than the increase among men.

The annual incidence rates of lung cancers overall by subtype and sex are presented in Figure 1B and the data are listed in Supplementary Table 1, http://links.lww.com/MD/A300. The most common subtype of lung cancers was adenocarcinoma, followed by squamous cell carcinoma and small cell carcinoma for both men and women. The incidence rate of adenocarcinoma increased for both men (9.9 per 100,000 in 1996 to 18.5 per 100,000 in 2008, APC = 4.8, *P* < 0.0001) and women (7.6 per 100,000 in 1996 to 16.3 per 100,000 in 2008, APC = 5.9, *P* < 0.0001), although the increase was faster for women compared to men. By contrast, the annual incidence rate of squamous cell carcinoma did not change significantly for men (10.9 per 100,000 in 1996 to 10.5 per 100,000 in 2008, APC = -0.4, *P* = 0.32) and decreased significantly for women (2.1 per 100,000 in 1996 to 1.6 per 100,000 in 2008, APC = -2.7, *P* = 0.0001). For small cell lung cancer, the annual incidence rate increased significantly among men (3.9 per 100,000 in 1996 to 5.4 per 100,000 in 2008, APC = 2.8, *P* < 0.0001) but not among women (0.5 per 100,000 in 1996 to 0.6 per 100,000 in 2008, APC = 1.0, *P* = 0.33). The rare subtypes of lung cancer included large cell carcinoma, sarcoma, lymphoma and NET. Large cell carcinoma accounted for 0.62% of lung cancers and was the fourth most common subtype of lung cancer. It was more common in men than women. The annual incidence increased significantly for men (0.19 per 100,000 in 1996 to 0.34 per 100,000in 2008, APC = 4.3, *P* = 0.007) but not for women (0.07 per 100,000 in 1996 to 0.11 per 100,000 in 2008, APC = 0.6, *P* = 0.78). Sarcoma accounted for 0.19% of all lung cancers and the change in annual incidence was not significant for either men (0.06 per 100,000 in 1996 to 0.08 per 100,000 in 2008, APC = 3.9, *P* = 0.08) or women (0.05 per 100,000 in 1996 to 0.02 per 100,000 in 2008, APC = −1.1, *P* = 0.84). Lymphoma accounted for 0.24% of all lung cancers. A significant increasing trend in the incidence of lung lymphoma was seen in women (0.01 per 100,000 in 1996 to 0.06 per 100,000 in 2008, APC = 9.0, *P* = 0.04) but not in men (0.07 per 100,000 in 1996 to 0.09 per 100,000 in 2008, APC = 4.0, *P* = 0.11). NET accounted for 0.44% of all lung cancers and a significant increase in the incidence rate was noted for NET in both men (0.05 per 100,000 in 1996 to 0.30 per 100,000 in 2008, APC = 17.9, *P* < 0.0001) and women (0.03 per 100,000 in 1996 to 0.08 per 100,000 in 2008, APC = 10.5, *P* = 0.003).

### Distributions and Incidence Trends of Lung Cancer by Age, Subtype, Sex, and Time Period

Because the changes in incidence rates of lung cancers were variable among the different subtypes and between sexes, the distributions of lung cancers by subtype, sex, and age group were evaluated in 3 time periods, 1996–1999 (T1), 2000–2004 (T2), and 2005–2008 (T3) (Supplementary Table 2, http://links.lww.com/MD/A300). The most common age of diagnosis was between 70 and 80 years old for all 3 time periods (34.8% in T1, 37.1% in T2, and 34.0% in T3). The second most common age of diagnosis was between 60 and 70 years old with a percentage of 30.9% in T1, 25.5% in T2, and 22.21% in T3. The percentage of lung cancer patients diagnosed at age >80 years increased gradually from 11.07% in T1 to 14.85% in T2 and to 18.63% in T3. The percentage of patients diagnosed at age > 60 years was 76.76%, 77.41%, and 74.81% in T1, T2, and T3, respectively. The age distribution pattern was similar in men and women. The proportion of female lung cancer cases increased with time and accounted for 30.25%, 32.07% and 34.4% of all lung cancers in T1, T2, and T3, respectively.

The distributions of lung cancers by subtypes and sexes in the 3 time periods are shown in Figure 1C and Supplementary Table 2, http://links.lww.com/MD/A300. Adenocarcinoma was the most common subtype of lung cancer for both men and women and the percentage increased with time. The percentage of adenocarcinoma in men increased from 31.1% in T1 to 36.1% in T2 and to 40.7% in T3. The percentage of adenocarcinoma in women increased from 53.9% in T1 to 62.9% in T2 and to 70.9% in T3. In contrast, the percentage of squamous cell carcinoma, which was the second most common subtype of lung cancer, decreased with time in both men and women. The percentage of squamous cell carcinoma in men was 30.87%, 28.70%, and 25.90% in T1, T2, and T3, respectively, while the percentage in women was 12.53%, 9.68% and 7.76% in T1, T2, and T3, respectively. The distribution of small cell carcinoma was not significantly different in the 3 time period for men (10.9% in T1, 11.62% in T2, and 12.01% in T3) and women (3.08% in T1, 3.48% in T2, and 2.69% in T3). Although smoking is a major risk factor for both small cell carcinoma and squamous cell carcinoma of the lung, no declining trend in the percentage of small cell carcinoma was observed, in contrast to the declining trend in the percentage of squamous cell carcinoma.

### The Stage Distribution of Lung Cancer by Subtypes and Sexes

To learn about the distribution of early and advanced lung cancer patients, we evaluated the stage at diagnosis by subtypes and sexes. The proportion of unknown stage was 99.71%, 74.77%, and 20.99% in T1, T2, and T3, respectively; therefore, we decided to only examine the distribution of stage in T3 (Table 1). Approximately 18% of patients were diagnosed at early stages (stages 0, I, and II) for adenocarcinoma and squamous cell carcinoma. Approximately 23% of large cell carcinoma and 30% of NET were also diagnosed at early stages. In contrast, only 6% of small cell carcinoma cases were diagnosed at early stages. For lung adenocarcinoma and NET, more women were diagnosed at early stages than men. In contrast, more men were diagnosed at early stages than women for lung squamous cell carcinoma. There were no significant difference in the distribution of stage by sexes for small cell carcinoma and large cell carcinoma. We did not include the stage distribution for lymphoma and sarcoma because >50% of stage information for lymphoma and sarcoma were missing.

**Table 1:**
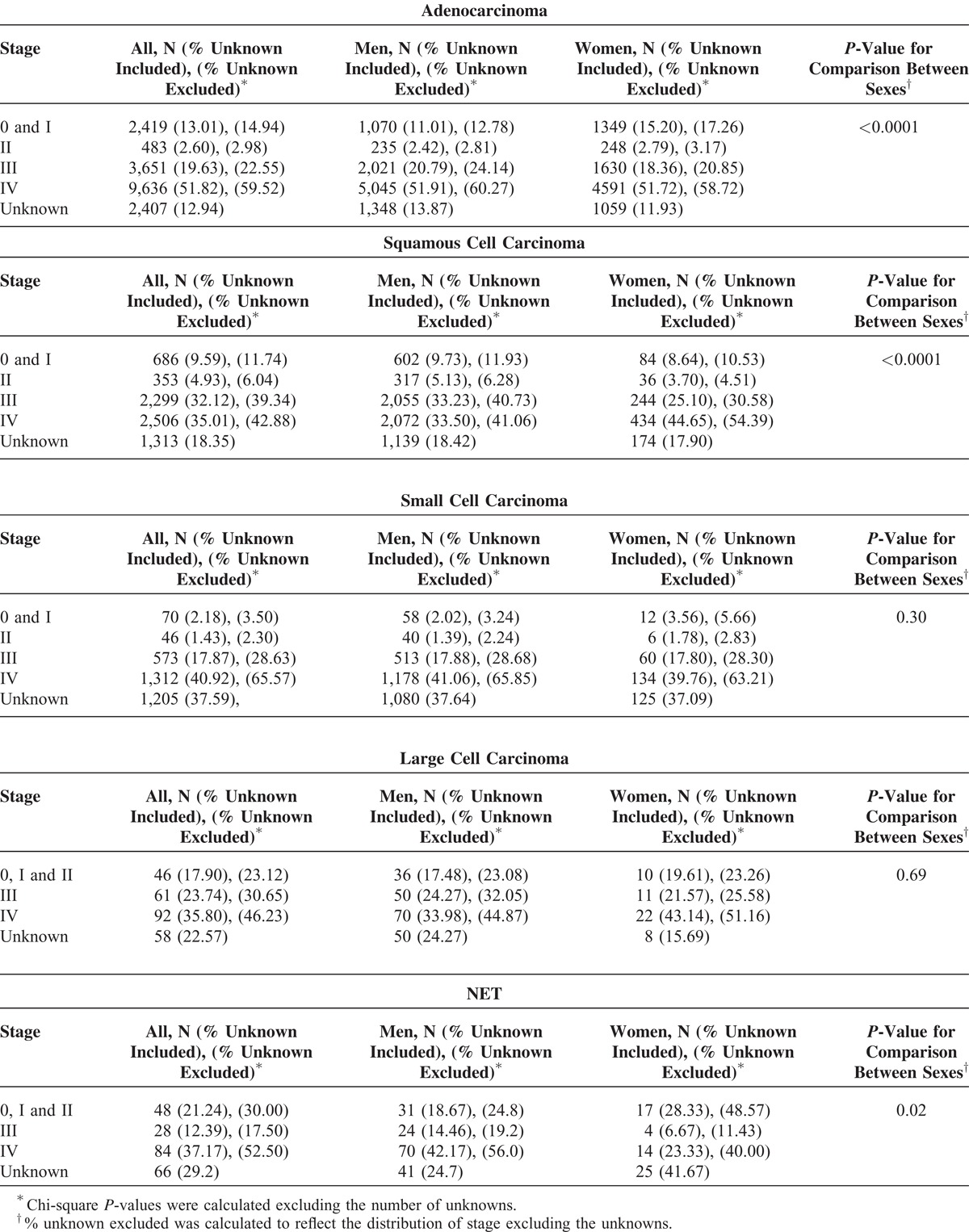
The Stage Distribution of Incident Lung Cancer Cases in Taiwan Between 2005 and 2008 by Subtype and Sex

### Survival of Lung Cancer Patients by Subtype, Sex, and Time Period

**FIGURE 2:**
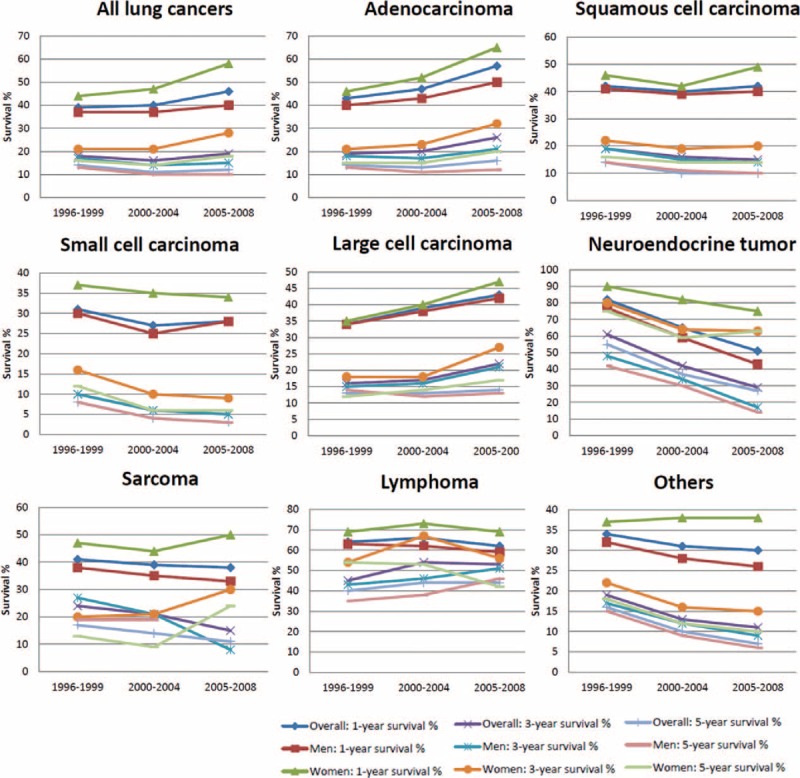
Survival probability of lung cancer patients at 1, 3 and 5 years by subtype, sex, and time period of diagnosis.

The survival of lung cancer by subtype, sex, and the 3 time periods is presented in Table 2  and Figure 2. The 1-, 3-, and 5-year survival probabilities for all lung cancer patients during 1996–2008 were 41%, 18%, and 13%, respectively. Generally, the survival was better for women than for men. The 1-, 3-, and 5-year survival probabilities were 37%, 15 %, and 11%, respectively, for men and 50%, 24%, and 16%, respectively, for women. The survival curves by subtypes for both sexes combined and for each sex separately are shown in Supplementary Figure 1, http://links.lww.com/MD/A299. Among the subtypes, the best survival was seen in patients with lymphoma, followed by NET and adenocarcinoma. Patients with small cell carcinoma had the worst survival probabilities. For women, the best survival probability occurred in NET, followed by lymphoma and adenocarcinoma. In contrast, the best survival probability for men was for lymphoma, followed by NET. The survival probabilities among subtypes were variable in the 3 time periods (Table 2  and Figure 2). A significant improvement in the survival probability was noticed in the recent time period (2005–2008) for adenocarcinoma, more significantly for women than men. For men, the 1- and 3-year survival probability of adenocarcinoma increased by 10% and 3%, respectively, from T1 to T3, whereas the 1-, 3-, and 5-year survival probability of adenocarcinoma for women during T1 to T3 increased by 18%, 11%, and 5%, respectively. The survival probability in men with squamous cell carcinoma became worse from T1 to T3, but for women, the survival probability of squamous cell carcinoma did not change significantly. For small cell carcinoma, the survival probability of male patients became worse from T1 to T3. Decreased survival probabilities were also seen in female patients with small cell carcinoma, but they were not statistically significant. The survival probabilities of large cell carcinoma, sarcoma, and lymphoma were not significantly different in the 3 time periods. The survival probability of NETs became worse from T1 to T3 for both men and women. Taken together, during 1996–2008, improvement in survival was seen in patients with adenocarcinoma, particularly for women, and the survival probability became worse for small cell carcinoma and NET among all patients and for squamous cell carcinoma among men.

**TABLE 2:**
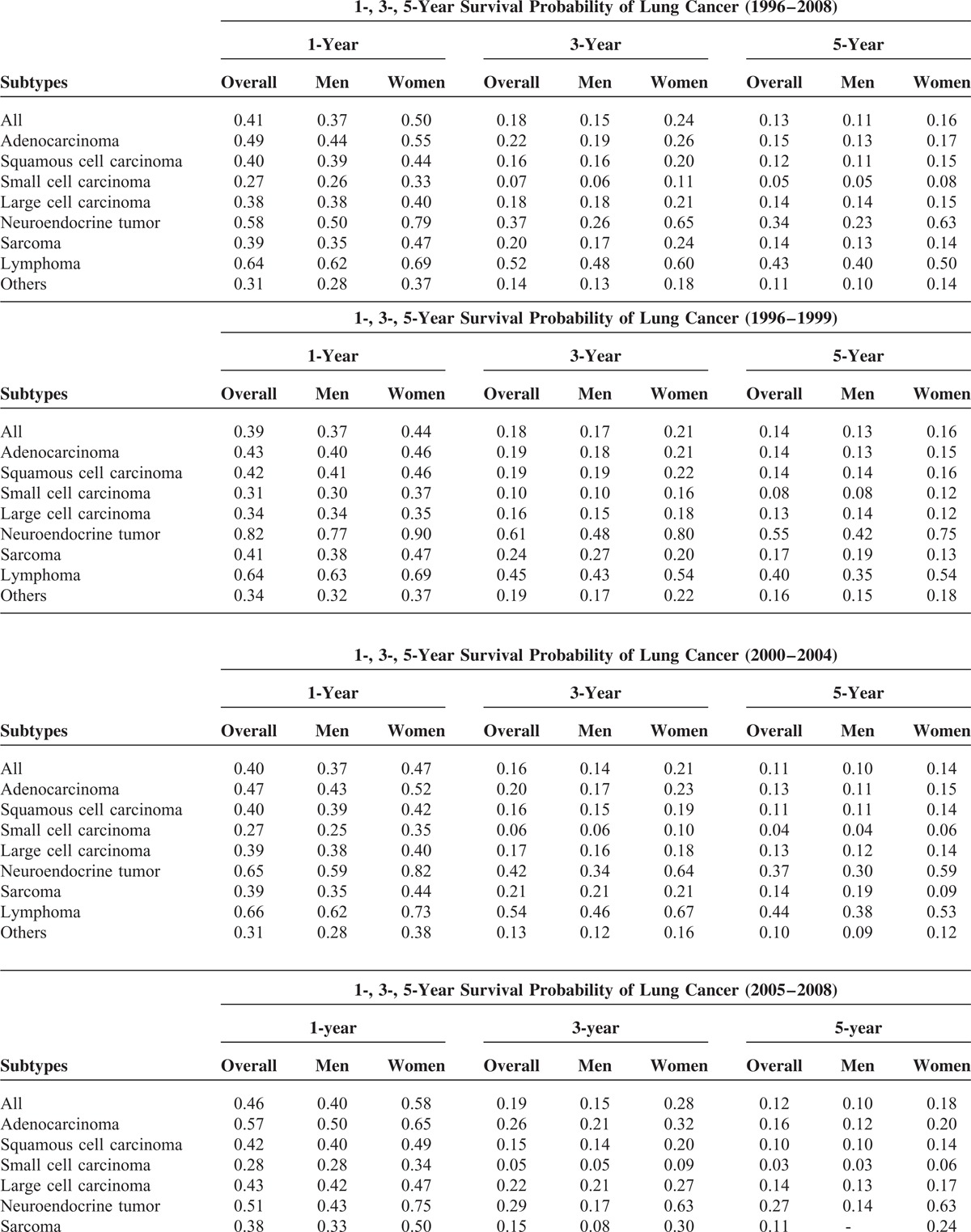
Survival Probability of Lung Cancer Patients at 1, 3 and 5 Years by Subtype, Sex, and the Time Period of Diagnosis^∗^

**TABLE 2 (Continued):**
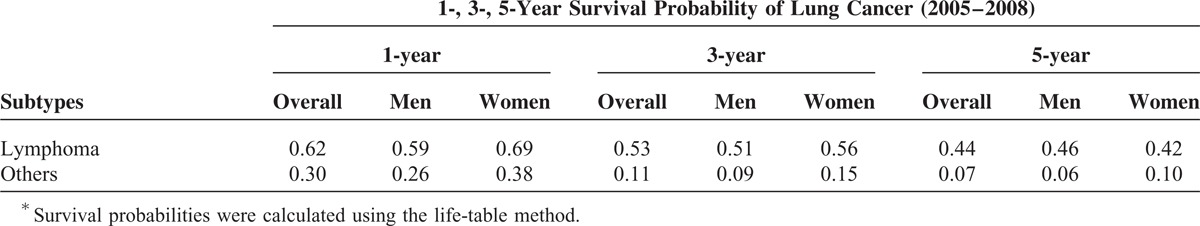
Survival Probability of Lung Cancer Patients at 1, 3 and 5 Years by Subtype, Sex, and the Time Period of Diagnosis^∗^

The results of the Cox proportional hazards survival analysis by subtype, sex, age, and diagnosis periods are presented in Table 3. Adenocarcinoma, the most common lung cancer subtype, was used as the referent for subtype analysis. NET and lymphoma had a significantly better prognosis than adenocarcinoma in the univariate analysis. The significance was still present in the multivariate analysis for NET (HR = 0.68, 95% CI: 0.61–0.76) and for lymphoma (HR = 0.54, 95% CI: 0.45–0.63), adjusted for sex, age and the diagnosis period. The survival probability in women was significantly better than in men (HR = 0.83, 95% CI: 0.82–0.84) in multivariate analysis. The prognosis of lung cancer patients grew poorer with increasing age, with those aged 80 years and older having the worst prognosis. We used T1 as the reference group to evaluate whether the more recently diagnosed lung cancer cases (those diagnosed in T2 and T3) might have a better prognosis because of the availability of the newer therapeutic agents and improvement in supportive care for lung cancer patients. Overall, the survival of lung cancer in T3 was slightly better than in T1 (HR = 0.98, 95% CI: 0.96–0.99) according to the multivariate analysis. When stratified by sexes, the improvement in survival probability during T3 compared to T1 was only observed among women (HR = 0.85, 95% CI: 0.82–0.88) and not among men. Table 4 presents the Cox proportional hazards analysis for the survival probabilities of lung cancers by subtypes, sexes, and age for the 3 time periods and by stages for T3 only. The prognosis of squamous cell carcinoma was better than adenocarcinoma in T1 (HR = 0.94, 95% CI: 0.91–0.98) but became worse than adenocarcinoma in T3 (HR = 1.03, 95% CI: 1.00–1.06), which indicated the improvement in the survival of adenocarcinoma patients. When compared to men, the risk of death in women was consistently lower and the difference became more significant with time; the HR decreased from 0.91 (95% CI, 0.88–0.93) in T1 to 0.86 (95% CI, 0.84–0.88) in T2 and T3. The risk of death was also affected by stages with higher stages being associated with a poorer survival.

**TABLE 3:**
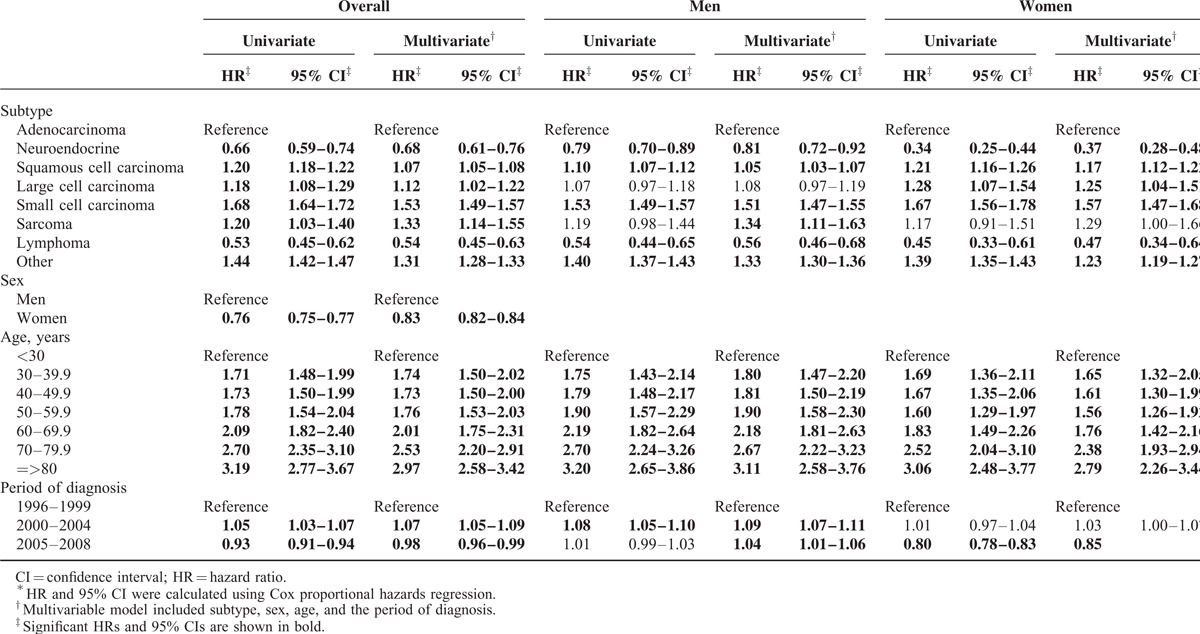
Cox Proportional Hazards Regression Analysis of Lung Cancer, 1996–2008^∗^

**TABLE 4:**
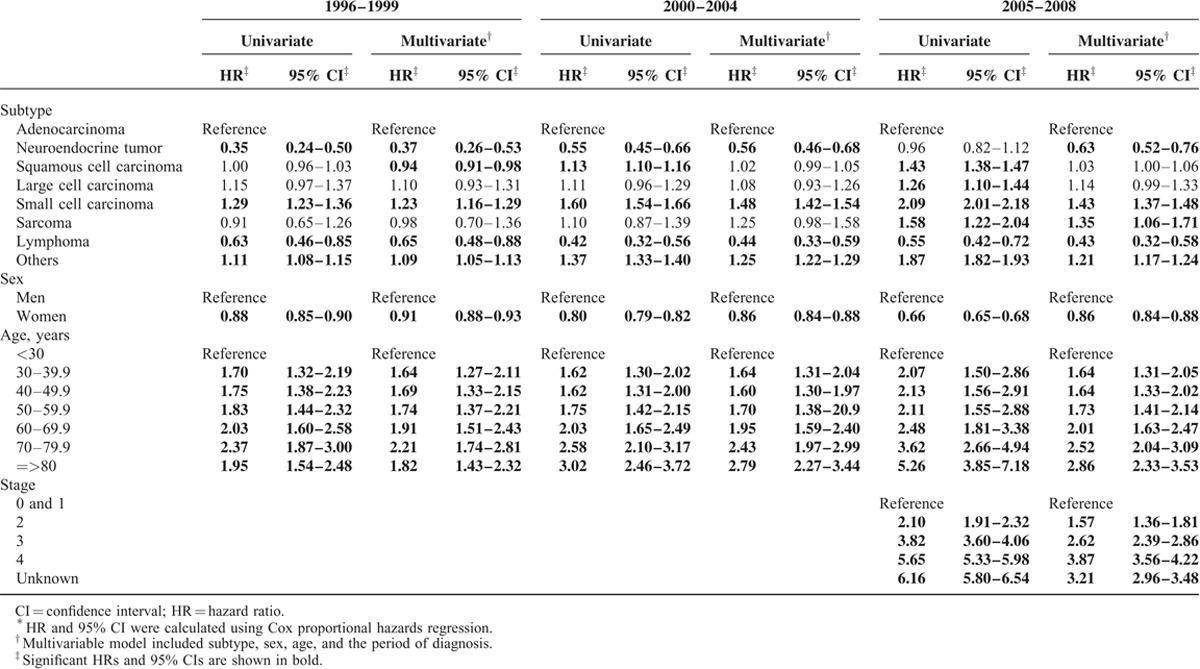
Cox Proportional Hazards Regression Analysis of Lung Cancer by the Time Period of Diagnosis^∗^

Because the prognoses among the subtypes of lung cancers were variable, we analyzed the risk of death by each subtype separately (Table 5 ). Among patients with adenocarcinoma, squamous cell carcinoma, small cell carcinoma, and NET, women had a longer survival than men. The risk of death for large cell carcinoma, lymphoma, and sarcoma patients was not significantly different between men and women. Generally, patients diagnosed at an older age had a worse survival probability than those diagnosed at younger age, except for patients with large cell carcinoma. When compared with the survival probability of lung adenocarcinoma patients in T1, improved survival was noted for adenocarcinoma patients diagnosed in T2 (HR = 0.98, 95% CI: 0.95–1.00) and T3 (HR = 0.80, 95% CI: 0.77–0.82). The survival probabilities of squamous cell carcinoma and small cell carcinoma patients in T2 and T3 were slightly worse than those in T1. The risk of death for patients with large cell carcinoma and sarcoma was not significantly different in the 3 time periods. The survival probabilities of NET patients became worse in T3 (HR = 2.05, 95% CI: 1.34–3.13) when compared with the survival of NET patients in T1. For NETs diagnosed in T1, 78.4% were carcinoid tumors and 21.6% were neuroendocrine carcinomas. In contrast, only 30.1% and 23.45% of NETs in T2 and T3, respectively, were carcinoid tumors. Increased percentages of poor-prognostic subtypes of NET were noticed in T2 and T3. Atypical carcinoid accounted for 7.19% and 5.75% of pulmonary NET in T2 and T3, respectively. Large cell neuroendocrine carcinoma accounted for 20.3% of pulmonary NET in both T2 and T3, respectively. Neuroendocrine carcinoma accounted for 41.2% and 35.4% of pulmonary NET in T2 and T3, respectively. The decrease in the percentage of carcinoid tumors in men (74.2% in T1, 21.9% in T2, and 10.2% in T3) was more prominent than the decrease in women (85% in T1, 53.8% in T2, and 60.0% in T3). Taken together, the overall survival probability was significantly prolonged in the last decade for patients with adenocarcinoma, but reduced survival was found in patients with NET.

**TABLE 5:**
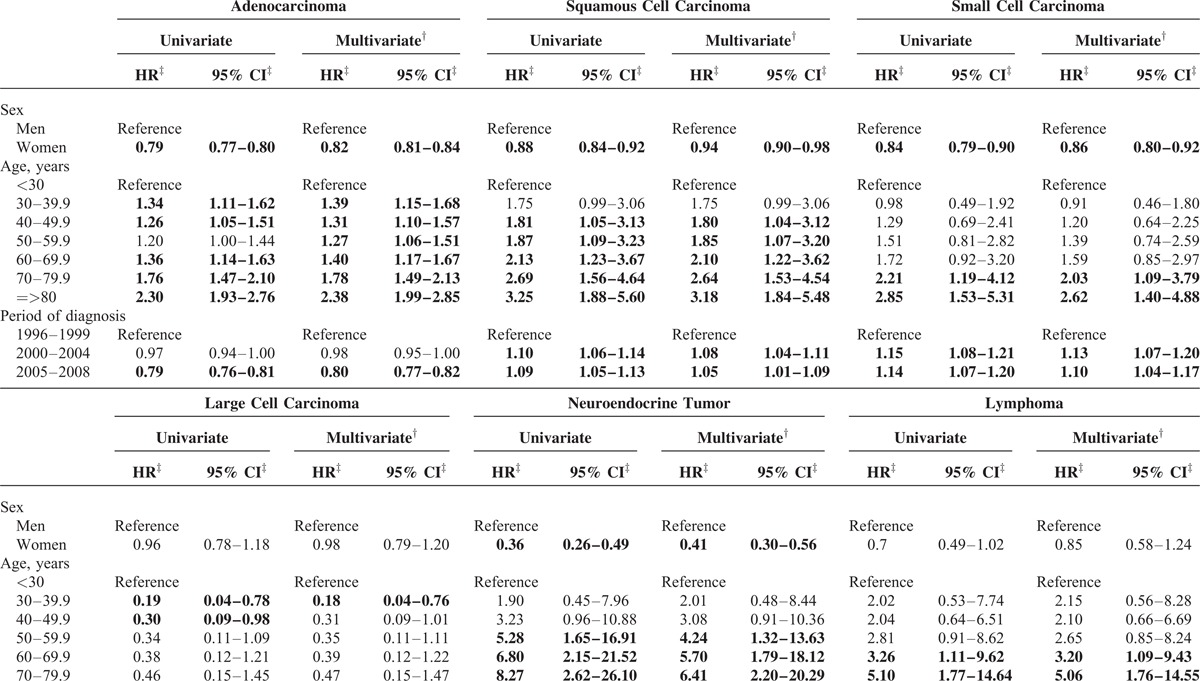
Cox Proportional Hazards Regression Analysis of Lung Cancer by Subtype^∗^

**TABLE 5 (Continued):**
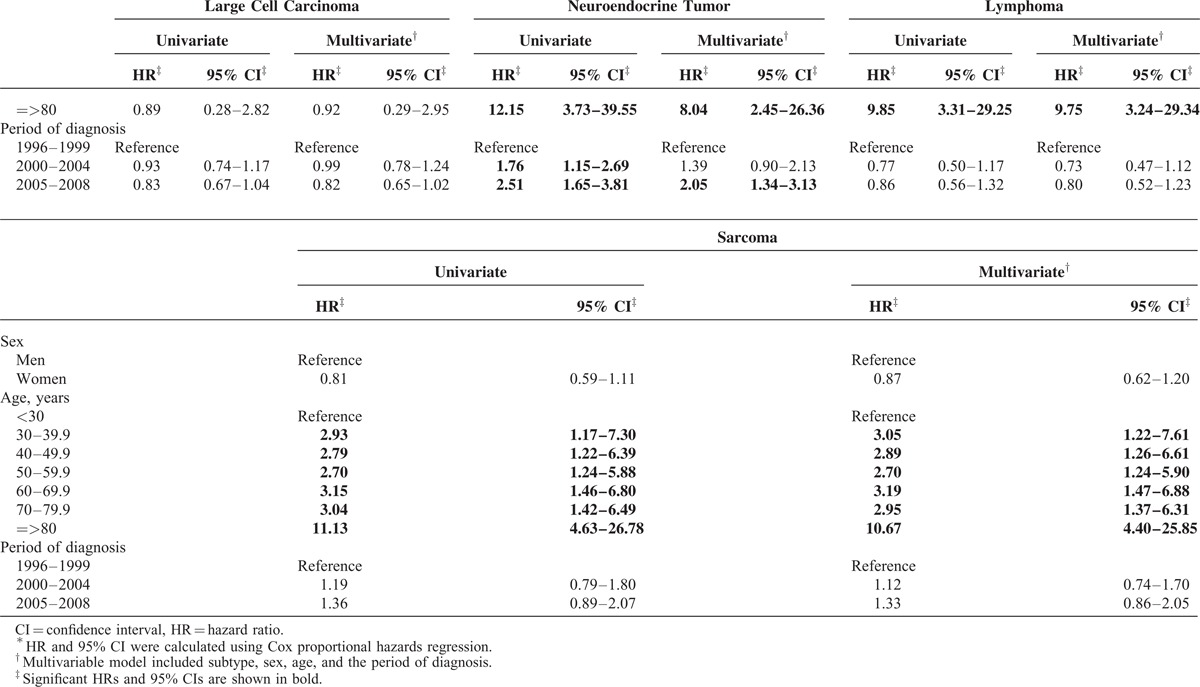
Cox Proportional Hazards Regression Analysis of Lung Cancer by Subtype^∗^

## DISCUSSION

In this nation-wide population-based study, we observed several changes in the incidence and survival of lung cancer, including an increased incidence of adenocarcinoma and NET and an improved probability of survival for patients with lung adenocarcinoma and lymphoma. According to Lortet-Tieulent et al., the incidence rates of lung cancers changed markedly from the 1970s to 2000s; the direction and the magnitude of change varied by sex and histologic subtype.^3^ The age-standardized incidence rates among various countries in America, Europe, and Oceania stratified by sex ranged from 17.0 per 100,000 in Spanish women to 176.9 per 100,000 in US black men. Generally, men had a higher incidence rate of lung cancer than women in most countries except in Iceland (men: 73.6 per 100,000; women: 76.0 per 100,000). The male-to-female incidence ratio was approximately 2 in most countries, but Spain (men: 120.6 per 100,000, women: 17.0 per 100000, ratio = 7.1) and France (male: 124.8 per 100,000, women: 22.7 per 10,000, ratio = 5.5) had a particularly high proportion of lung cancer occurring in men. Generally, lung cancer incidence rates have been decreasing overall and for all subtypes among men except for adenocarcinoma.^19^ The incidence rates of lung cancers have continued to increase among women and the increase has been particularly prominent for adenocarcinoma.^19^ Using data from the Osaka Cancer Registry in Japan, Toyoda et al^23^ reported that the incidence rate of lung cancer among Japanese men increased from 1975 (46.2 per 100,000) to late 1980s and then remained stable from 1989 to 2003 (approximately 65 per 100,000). In contrast, the incidence rate of lung cancer in Japanese women continued to increase from an incidence rate of 12.9 per 100,000 in 1975–1978 to 20.2 per 100,000 in 1999–2003.^23^ The male-to-female lung cancer incidence ratio in Japan was 3.2 from 1999 to 2003.^23^ Using data from the Korean Cancer Registries in South Korea, Lee et al^24^ reported that the incidence rate of lung cancer increased from 44.8 per 100,000 to 51.3 per 100,000 in Korean men and from 6.7 per 100,000 to 13.3 per 100,000 in Korean women during 1993-1998. The more rapid increase in the incidence of lung cancer among Korean women compared to that of Korean men was reflected in a decrease of male-to-female ratio of lung cancer incidence from 6.7 in 1993 to 3.9 in 1998. Generally, the male-to-female lung cancer incidence ratio has been approximately 2 to 3 in Asian populations. Gomez et al^25^ also reported a similar male-to-female lung cancer incidence ratio among Asian American populations in the SEER database. Similar to Japan and Korea, the overall lung cancer incidence in Taiwan has been increasing at a faster rate for women than for men. The male-to-female ratio of lung cancer incidence in Taiwan decreased from 2.1 in 1996 to 1.9 in 2008.

Adenocarcinoma is the most common subtype of lung cancer worldwide, particularly in women. Increasing trends in the incidence of lung adenocarcinoma have been observed in Western countries, Japan, and Taiwan with women experiencing a faster rise than men.^19,23^ Adenocarcinoma accounts for a higher proportion of lung cancer among Asian women than it does among women from Western countries. In Japan, 58.7% to 67.2 % of lung cancer cases diagnosed in women during 1975–2003 were adenocacinoma.^23^ In our study, adenocarcinoma accounted for 64% of lung cancer cases in Taiwanese women. In contrast, the proportion of adenocarcinoma in lung cancer among Western women ranged from 27% to 54%, with the lowest being 27% in the Netherlands and the highest being 53.8% in Canada.^3^. The higher percentage of adenocarcinoma among Asian women with lung cancer could be partly explained by shared genetic background and environmental risk factors; however, further investigations are needed to explain this phenomenon. Smoking is a major cause of lung cancer, particularly for squamous cell carcinoma and small cell carcinoma. A globally decreasing trend of lung squamous cell carcinoma concurrent with an increasing trend of adenocarcinoma indicates the contribution of other risk factors to the development of adenocarcinoma. Exposures to cooking fumes, air pollutions, and radon have been associated with an increased risk of lung adenocarcinoma.^26^ Other possible risk factors of lung adenocarcinoma include nutritional status, genetic susceptibility, immunologic dysfunction, tuberculosis, asthma, and human papillomavirus (HPV) infection.^26^ Other than adenocarcinoma, a rapid increase in NET and lymphoma was also noted in our study. The increase in the incidence of lung NET could be partially attributed to the establishment of pathologic classification and increased awareness of this disease. An increasing trend of lung NET has also been reported by studies from USA and Norway, but the etiologies are yet to be elucidated.^22,27^ The incidence of lung lymphoma also increased for both Taiwanese men and women. The increasing trend in the incidence of lung lymphoma may be explained by the increase in the overall incidence rate of lymphoma in Taiwan. The overall incidence rate of lymphoma increased among Taiwanese men from 5.87 per 100,000 in 1996 to 8.1 per 100,000 in 2008 (APC = 3.8) and among Taiwanese women from 3.77 per 100,000 in 1996 to 5.69 per 100,000 in 2008 (APC = 4.3).^2^

In our analysis, we observed an improvement in the survival of patients with lung adenocarcinoma over the years, which may be attributed to the introduction of EGFR inhibitors. Gefitinib, an EGFR tyrosine kinase inhibitor, has been found to induce responses among patients who were more likely to be Asian, women, nonsmokers, and diagnosed with NSCLC. This observation was later correlated with EGFR mutations. In a large multinational series of NSCLC, the prevalence of EGFR mutation was 27% and 34% in Taiwanese and Japanese NSCLC patients, respectively, and only 14% and 7% in US and Australian NSCLC patients, respectively.^11^ A percentage of EGFR mutations as high as 61% was noted in Taiwanese samples from clinical trials.^10,12,28^ EGFR inhibitors not only increased the response rate of lung cancers with EGFR mutations but also prolonged the progression-free survival and overall survival. In Taiwan, Gefitinib was approved as a third-line treatment for lung adenocarcinoma in 2003, and the reimbursement for such use was approved by the Bureau of National Health Insurance (BNHI) in November 2004. Since November of 2007, the BNHI began to reimburse Gefitinib as a second-line treatment for adenocarcinoma and in June of 2011, the BNHI started to reimburse Gefitinib as a first-line treatment for lung adenocarcinoma with EGFR mutations. Another EGFR inhibitor, Erlotinib, was also reimbursed by the BNHI for treating NSCLC since 2007. The use of EGFR inhibitors for lung adenocarcinoma since 2004 may explain the improvement in the survival of adenocarcinoma patients from 2000–2004 to 2005–2008, particularly for women, with improvement of the 1-, 3- and 5-year survival from 46.24%, 20.86%, and 15.26% in 1996–1999 to 64.53%, 31.81% and 19.86% in 2005–2008, respectively. In a large molecular epidemiologic study of lung adenocarcinoma in Asian patients, including patients from Taiwan, women were found to have a significantly higher percentage of EGFR mutations than men (61.1% vs 44%).^29^ The higher percentage of EGFR mutations among female patients with lung adenocarcinoma compared to men with lung adenocarcinoma may explain the better survival of lung adenocarcinoma in Taiwanese women, particularly after the introduction of EGFR inhibitors.

Among the rare tumors of pulmonary origin, NET patients exhibited the best survival. The major treatment for NET is surgery in its early stage and the prognosis for pulmonary NET depends on the subtype in addition to the stage. The best 5-year survival is in NET patients with typical carcinoid (78%–97%), followed by atypical carcinoid (35–75%), and neuroendocrine carcinoma (<50%).^30–36^ The decreased percentage of carcinoid tumors in our NET population, particularly for men, may explain the worsening survival in the later diagnosis years. It is not clear why the distribution of NET subtypes changed over time. One possible reason is that after the revision of NET classification by the World Healthcare Organization in 2000, physicians became more aware of NET, resulting in more cases of NET being diagnosed.^37^ Before 2000, patients with atypical carcinoid, neuroendocrine carcinomas, or large cell neuroendocrine carcinomas could possibly be misdiagnosed as carcinoma or other types of lung cancer due to lack of diagnostic tools before 2000. In addition, the significant reduction in the survival of male NET patients over time may result from the marked decrease in the proportion of carcinoid tumors, which occurred to a lesser extent among female NET patients. Thus, the reduction in the survival of male NET patients could likely be attributed to the more accurate diagnosis of NET in the later periods.

This study has several limitations. First, data in the TCR do not contain variables on lifestyle factors, including cigarette smoking; therefore, we could not account for the influence of cigarette smoking in our analysis. Second, although we observed differences in the stage distributions of lung cancer subtypes (adenocarcinoma, squamous cell carcinoma, and NET), we could not determine the causes for these differences using the data from the cancer registry. Third, while our results are representative of the Taiwanese population, they may not be generalized to other Asian populations. Finally, although we analyzed the survival probability of lung adenocarcinoma before and after the introduction of EGFR inhibitors, this does not prove a direct link between the use of EFGR inhibitors and the improved survival of lung adenocarcinoma, only a possible association between the 2 variables. In summary, the incidence of lung cancer in Taiwan, particularly that of adenocarcinoma in women, has been increasing rapidly. The introduction of targeted therapies was associated with a significantly improved survival of lung adenocarcinoma in Taiwan. More studies are needed to explain the rising incidence of lung adenocarcinoma in order to establish effective preventive strategies. In addition, it is important to investigate the molecular pathogenesis of various subtypes of lung cancer in order to develop novel therapeutic agents.
